# Morphological and anatomical evidence support a new wild cassava: *Manihot fallax* (Crotonoideae, Euphorbiaceae), from Mato Grosso, Brazil

**DOI:** 10.3897/phytokeys.91.21465

**Published:** 2017-12-19

**Authors:** Marcos José da Silva, Laís de Souza Inocencio, Rodolfo Carneiro Sodré, Alexandre Antonio Alonso

**Affiliations:** 1 Laboratório de Morfologia e Taxonomia Vegetal, Universidade Federal de Goiás, CP 131, 74001-970, Goiânia, GO, Brazil; 2 Postgraduate Student, Postgraduate Program in Plant Biodiersity, Universidade Federal de Goiás, CP 131, 74001-970, Goiânia, GO, Brazil; 3 Laboratório de Anatomia Vegetal, Universidade Federal de Goiás, CP 131, 74001-970, Goiânia, GO, Brazil

**Keywords:** Central Brazil, leaf anatomy, nomenclature, typification, Manihoteae

## Abstract

During the preparation of the taxonomic treatment of *Manihot* in the Midwest Region of Brazil, a new species was found. *Manihot fallax* M.J. Silva & L.S. Inocencio is described, illustrated and morphologically compared with similar simple-leaved species. The conservation status, geographic distribution (including map), ecology, phenology and notes about leaf anatomy of the new species are given. The synonymisation of *M. robusta* M. Mend. & T. B. Cavalc. under *M. attenuata* Müll. Arg. and lectotypes for *M. attenuata* and *M. brachystachys* Pax & K. Hoffm are also proposed. An emended description of *M. attenuata* is proposed as the original description is incomplete as it lacks information on the pistillate flowers, fruits and seeds.

## Introduction

The taxonomy of *Manihot* was studied by Rogers and Appan (1973), who recognised 98 species distributed into 19 sections. The genus is monophyletic and has a complex taxonomy (Duputié et al. 2011, Silva 2014). In Brazil, it is represented by over 80 species, especially growing in the Cerrado region, in rocky fields and grasslands (Silva 2014, Silva et al. 2017).

Despite the revision of *Manihot* (Rogers and Appan 1973), taxonomic studies about this genus in Brazil are scarce. *Manihot* is cited in floristic surveys of Euphorbiaceae (Cordeiro 1992, Sátiro and Roque 2008) or as an isolated genus (Allem 1997, Rodrigues 2007, Carmo-Júnior et al. 2013, Orlandini and Lima 2014). In the last four years, a number of species of *Manihot* have been described in Brazil in the coastal sand plains of the state of Sergipe (Martins et al. 2011), in the semi-arid region of the state of Bahia (Martins et al. 2014) and in Cerrado *sensu lato* of the state of Goiás (e.g. Mendoza et al. 2015, Silva et al. 2013, 2016, 2017, Silva 2014, 2015, Silva and Sodré 2014).

Considering the species of *Manihot* present in the Midwest Region of Brazil, the knowledge about the genus is scarce mainly in the states of Mato Grosso and Mato Grosso do Sul, where BFG (2015) reported 17 and 6 species respectively. However, taking into consideration that the flora of these states is relatively poorly known, that *Manihot* has its main diversity centre in the Brazilian Cerrado and also that these states have their areas covered predominantly by Cerrado vegetation, it is plausible to conclude that the number of species of the genus has been underestimated in Mato Grosso and Mato Grosso do Sul.

During the preparation of the taxonomic treatment of *Manihot* in the Midwest Region of Brazil, some collections were found from Serra Azul, Serra do Roncador, Serra do Taquaral and neighbouring areas in the state of Mato Grosso, which could not be assigned to any known species. One of these species, with entire and unlobed leaves and habit slender and virgate, is hereby illustrated, described as new and designated as *M. fallax*. The new species is compared with *M. attenuata* Müll. Arg. and *M. weddelliana* Baill., the taxa most morphologically similar to it and its phenology, conservation status and geographic distribution are also presented. The leaf anatomy of the new species and the species most morphologicaly similar to it were also compared because leaf anatomy constitutes an important tool for delimiting taxa in *Manihot* (e.g. Vannucci 1982, Cunha Neto et al. 2014, Cunha Neto et al. 2017 and Graciano-Ribeiro et al. 2016). Additionally, as part of these studies on *Manihot* of the Cerrado flora, *M. robusta* as a synonym of *M. attenuata* Müll. Arg. is proposed and *M. attenuata* and *M. brachystachys* Pax & K. Hoffm are lectotypified. An emended description of *M. attenuata* is provided, as the original description is incomplete as it lacks information on the pistillate flowers, fruits and seeds.

## Materials and methods

### Morphological and taxonomic studies

The morphological description of the new species is based on field observations, conducted by the authors during expeditions to the state of Mato Grosso and on morphological analyses of 34 collections from the authors and 10 collections from herbaria (UFMT/ICLMA and IAC). The emended description of *Manihot attenuata* and the nomenclatural revision for all taxa associated with it, resulted from analysis of all protologues, type and historical collections (17 exsiccatae) of the same and also of extensive field work in their areas of occurrence, as well as analyses of 30 collections from herbaria (BR, CEN, F, G, HRCB, HUEFS, IBGE, K, MG, MO, NY, P, RB, S, SP, UB and UFG). The terminology used in the description of both species is based on specific literature such as Pohl (1827), Pax (1910) and Rogers and Appan (1973); the last was employed mainly for the inflorescence types and venation pattern. All the samples used in the description of the new species, including the holotype, were deposited in the UFG herbarium and the isotypes will be sent to K, NY and MO. The acronyms of herbaria previously cited follow Thiers (2017, continuously updated)

The conservation assessment of both the species was based on field observations and applying the IUCN Red List Categories and Criteria (IUCN 2014). The geographic distribution map was made using the software QGIS (Quantum GIS Development Team) version 2.8.1, which was used for the geographic coordinates obtained both during the collection expeditions and from the labels of the collections examined. The extent of occurrence (EOO) was calculated with the Geospatial Conservation Assessment Tool (GeoCAT - http://geocat.kew.org), (Bachman et al. 2011).

### Anatomical studies

Leaves of the type collection of the new species (i.e. L. S. Inocencio, A. O. Souza, L. L. C. Antunes and C. C. Oliveira 293) and *M. attenuata* (M. J. Silva 4063/UFG) with fully expanded leaf blades were fixed in FAA (formaldehyde/glacial acetic acid/ethanol 50% at 1:1:18 v/v/v) for 48h and transferred to 70% ethanol (Johansen 1940). Freehand sections of the median portion of the leaf blade and petiole of the samples were cleared in sodium hypochlorite solutions (20%), rinsed with distilled water, stained with aqueous 1% astra blue and aqueous 1% Safranin (Bukastch 1972) and mounted in aqueous glycerine solution (1:1). For epidermal analysis, classification and observation of stomata distribution, leaf blade segments were dissociated using a hydrogen peroxide and acetic acid solution (Franklin 1945). Sections were examined and photographed using a Leica ICC50 camera coupled with a Leica DM500 microscope (Leica Microsystems, Heerbrugg, Switzerland).

## Taxonomic treatment

### Manihot fallax

Taxon classificationPlantaeMalpighialesEuphorbiaceae

M.J. Silva & L.S. Inocencio sp. nov.

urn:lsid:ipni.org:names:77174267-1

Figs 1 , 2

#### Type:

BRAZIL. Mato Grosso: Município de Barra do Garça, BR-158, na altura do km 726, margem esquerda da estrada no sentido Nova Xavantina, campo cerrado em encosta, 15°17'9.7"S, 52°11'15.5"W, 385 m a.s.l., 26 Jan 2014, fl., *L. S. Inocencio, A. O. Souza, L. L. C. Antunes, and C. C. Oliveira 293* (holotype: UFG; isotypes: K, MO, NY).

#### Diagnosis.

Shrubs slender and virgate, up to 1.9 m tall; leaves light green, entire, unlobed, with secondary veins perpendicular to the midvein. Stipules 1–3 × 0.2–0.3 mm, setaceous, entire, early caducous; racemes 2.5–6 cm long, solitary, subspicate, staminate buds obovoid, stamens with filaments pubescent, pistillate calyx dialisepalous and fruits light green, without wings.

**Figure 1.**
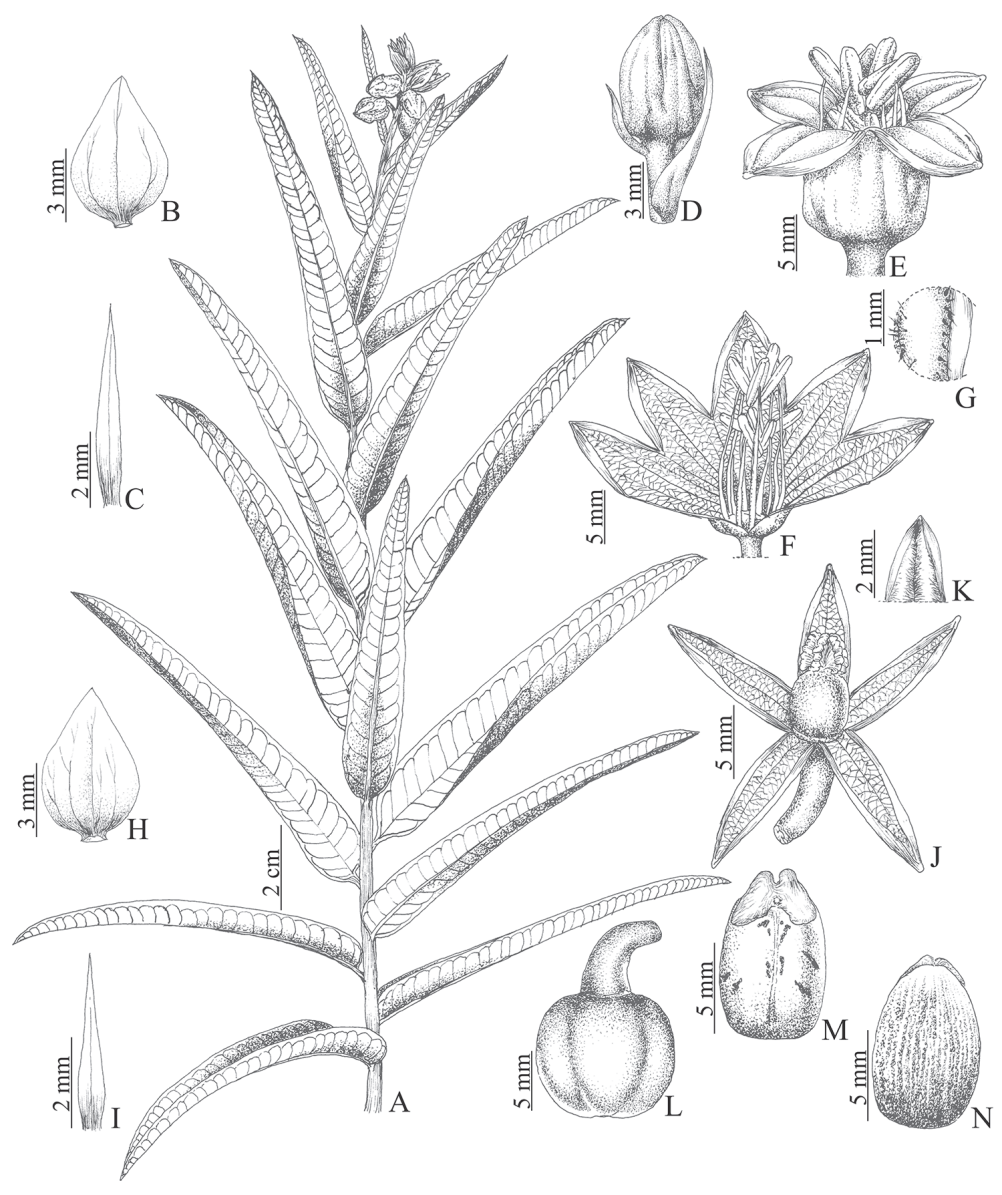
*Manihot fallax*. **A** Habit **B** Staminate bracts **C** Staminate bracteole **D** Staminate bud **E** Staminate flower **F** Staminate flower with calyx split and open **G** Inner surface of the staminate calyx showing the trichomes **H** Pistillate bracts **I** Pistillate bracteole **J** Pistillate flower **K** Inner surface of the pistillate calyx showing the trichomes **L** Fruit **M** Seed, ventral side **N** Seed, dorsal side. Drawn by Cristiano Gualberto from the holotype.

**Figure 2.**
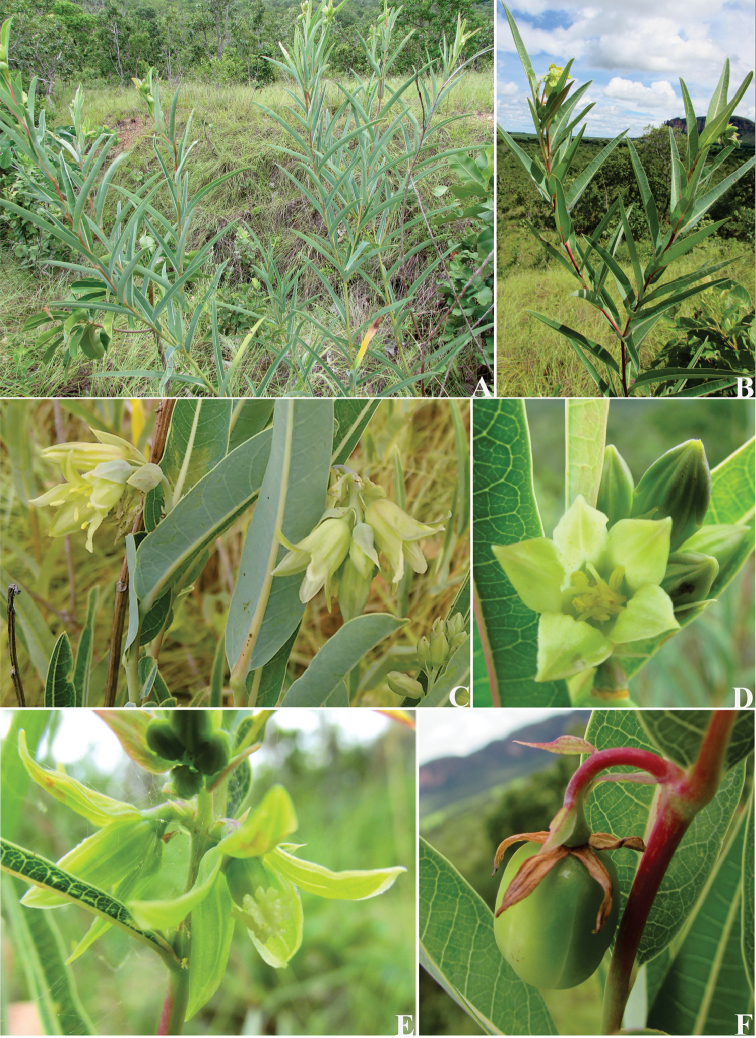
*Manihot fallax*. **A** Habit **B** Leaves; note the spiral phyllotaxy **C** Inflorescence; note the showy bracts **D** Staminate flower, frontal view **E**. Pistillate flowers in the inflorescence, lateral view; note the free sepals **F** Fruit.

#### Description.

Shrubs 1–1.9 m tall, slender and virgate, monoecious, glabrous. Stem solid in cross section, greenish to reddish, glossy and waxy in young parts; latex clear, abundant; roots fibrous. Stipules 1–3 × 0.2–0.3 mm, setaceous, margins entire, early caducous; petiole 0.3–0.7 mm long, angulate, greenish. Leaves alternate spiral, sparsely distributed along the branches, but more concentrated near apex, blades 8–14 × 1–2 cm, lanceolate, narrowly elliptic or linear, entire, unlobed, non-peltate, base obtuse, apex obtuse with a short mucro, firmly membranaceous to chartaceous, glabrous on both surfaces, adaxial surface dark green, abaxial surface glaucous, the latter with a smooth wax pattern, venation camptodromous-brochidodromous, primary and secondary veins impressed on both surfaces, the secondary veins perpendicularly arranged in relation to the midvein, all of them pinkish to yellowish. Racemes 2.5–6 cm long, staminate or bisexual, solitary, terminal or arising from dichotomy of the branches, glabrous, angulate, glaucous to cinereous, waxy. Staminate flowers: buds 5.8–6 × 3.9–4 mm, obovoid, yellowish-green, without purplish pigmentation; bracts 6–6.1 × 3.9–4 mm, widely elliptic, foliaceous, apex acuminate, persistent; bracteoles situated along the lower third up to half of the pedicel, 4–4.1 × 0.4–0.5 mm, lanceolate, foliaceous, persistent, subopposite; pedicels 0.5–0.7 mm long, cylindrical, glabrous, light green; calyx 13.2–14 × 5.9–6 mm, campanulate, yellowish, without purplish pigmentation, shortly tomentose internally, lobes widely triangular, ovate to oblong, apex obtuse, base truncate; stamens 10, in two whorls of five, filaments pubescent near apex, the longer 12.5–12.6 mm long, the shorter 8.4–8.5 mm long, both thickened; anthers 5–7 mm long, oblong, dorsifixed, bright yellow; disc 10-lobed, intrastaminal, dark yellow. Pistillate flowers: buds 6–9 × 4–6 mm, ovoid, green-yellowish, without purplish pigmentation; bracts 6.4–7 × 2.9–3 mm long, widely ovate, margin entire, glabrous; bracteoles 3.7–3.8 × 0.8–0.9 mm, lanceolate, foliaceous, persistent, margin entire, apex acuminate, opposite along the lower third of the pedicel, glabrous; pedicels 4–6 mm long, cylindrical-clavate, glabrous, green; sepals 10–12 × 5–7 mm, lanceolate, apex acute, shortly tomentose externally, yellowish, without purplish pigmentation; ovary 4–6 × 3–4 mm, globose to ovoid, glabrous, green, disc patelliform, lobed, yellow; styles 3, shortly united at the base, stigmatic surface 2–3 mm long, densely papillose. Capsules 0.9–1.5 × 0.8–1.2 cm, oblong, light green, smooth, glabrous, without wings, dehiscence septicidal and loculicidal; columella (carpophore) persistent, 0.9 × 2–2.2 mm (width at narrowest point in middle), narrowly alate. Seeds 0.7–1 × 0.39–0.4 cm, oblong-ellipsoid, dark grey, with black spots; caruncle triangular, prominent, apex bilobed, cream to yellowish.

#### Distribution and ecology.

**Figure 3.**
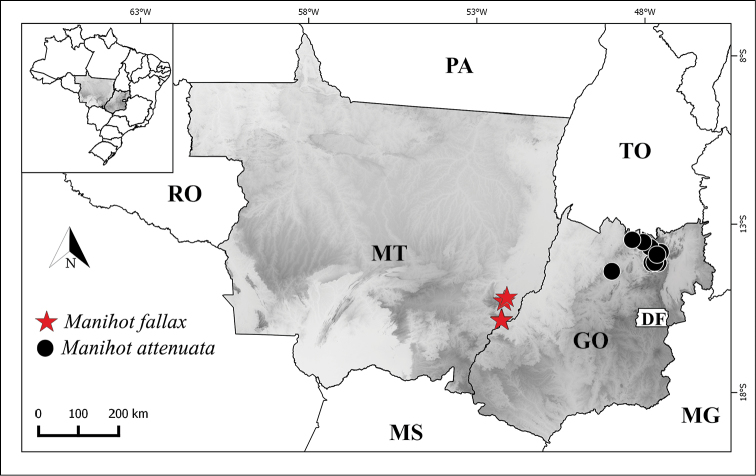
Distribution map of *Manihot fallax* sp. nov and *M. attenuata*.

*Manihot fallax* appears endemic to the state of Mato Grosso (Fig. 3), where it grows in Cerrado *sensu stricto* on flat or slope areas and also in grasslands, on clayey and sandy soils, between 385 m and 642 m elevation.

#### Phenology.

The species has been collected with flowers and fruits from October to March.

#### Etymology.

The specific Latin epithet “fallax” refers to deceptive and was chosen due to the false similarity of the new species to *M. attenuata* and *M. weddelliana*.

#### Conservation status.

According to the IUCN (2014), *M. fallax* can be considered preliminarily as an endangered species (EN-B2a), because it grows in disturbed areas subject to fires and human pressure and has an extent of occurrence of 175 km^2^.

#### Specimens Examined.

**Brazil. Mato Grosso**. Barra do Garças, Serra Azul, trilha as usina, próximo a sede da Moçonaria, 17 Mar 1990, fl., fr., *L. Santos s.n.* (UFMT/ICLMA 00856); próxima da cidade, nas imediações da antena do Cristo, 09 Mar 1991, fl., fr., *L. Santos s.n.* (UFMT/ICLMA 00866); Serra do Taquaral, Cerrado, 23 Nov 1997, fl., fr., *L. C. Bernacci & G. Arborcz 2525* (UFMT, IAC); cerrado do Marcello, 15°50'50.9"S, 52°15'54.7"W, 14 Nov 2005, fl., fr., *I. Faria & M. L. Mayer 339* (UFMT/ICLMA); gruta do pezinho, 15°50'29.6"S, 52°16'50"W, 28 Dec 2005, fl., *I. Faria, M. L. Mayer & Moisés 517* (UFMT/ICLMA); Parque Estadual da Serra Azul, 15°51'S, 52°16'W, 21 Oct 2005, fl., *M. Sanchez 2216* (UFMT/ICLMA); *ib.*, 21 Feb 2006, fl., fr., *I. Faria*, *M. L. Mayer & M. S. M. Ramos* 787 (UFMT/ICLMA); *ib.*, 15°52'S, 52°16'W, 04 Oct 2006, fl., *R. Freitas & M. L. Mayer 145* (UFMT/ICLMA); *ib.*, 13 Feb 2007, fl., *R. Freitas & M. L. Mayer 404* (UFMT/ICLMA); *ib.*, 1 Mar 2007, fl., fr., *R. Freitas & M. L. Mayer 445* (UFMT/ICLMA); BR 158 na altura do km 726, margem esquerda, sentido Nova Xavantina, adjacências da Serra do Roncador, 15°17'21.8"S, 52°11'38.7"W, 389 m a.s.l., 28 Jan 2014, *L. S. Inocencio, A. O. Souza & G. H. Silva 68, 69, 70*, *71*, *72* and *73* (UFG); *ib.*, 15°17'9.7"S, 52°11'15.5"W, 385 m a.s.l., 26 Jan 2015, fl. fr., *L. S. Inocencio, A. O. Souza, L. L. C. Antunes & C. C. Oliveira, 290, 291, 292* and *294* (UFG); BR 158, na altura do km 726, margem esquerda, sentido Nova Xavantina, 15°17'9.8"S, 52°11'22.3"W, 422 m a.s.l., 1 Feb 2015, fl. fr., *L. S. Inocencio, A. O. Souza, L. L. C. Antunes & C. C. Oliveira 363, 364, 365, 366, 367, 368, 369, 370, 371, 372, 373, 374, 375, 376, 377, 378, 379, 380, 381, 382, 383, 384, 385, 386, 387* and *388* (UFG); Serra do Roncador, ca. 60 km a norte da cidade pela BR-158 e 5 km a esquerda desta rodovia em direção a Xavantina a partir do povoado Vale dos Sonhos, 52°13'54"S, 15°20'54"W, 642 m a.s.l., 24 Mar 2016, *R. C. Sodré, T. P. Mendes & J. A. Oliveira 2174* (UFG).

#### Discussion.

*Manihot fallax* stands out from the other species of the genus with entire and unlobed leaves (Silva 2015) by its shrubby, slender, virgate habit up to 1.9 m tall, leaves ascendant and in a spiral arrangement along the branches. It morphologically resembles *M. weddelliana* and *M. attenuata*, especially the latter, in the aspect of the leaves, racemes conspicuously pedunculate, bracts of flowers of both sexes showy and foliaceous and calyx internally shortly tomentose. However, *M. fallax* differs from *M. weddelliana* in its shrubby, slender and virgate habit (vs. subshrub up to 0.5 cm tall in *M. weddelliana*), leaves without repand margins (vs. leaves with repand margins) and bracts of both staminate and pistillate flowers ovate, with margins entire and apex acute or obtuse (vs. widely elliptic, with margins very serrated and apex conspicuously acuminate). The combination of the characters listed in table below serve to differentiate *M. attenuata* from *M. fallax*.

**Figure 4.**
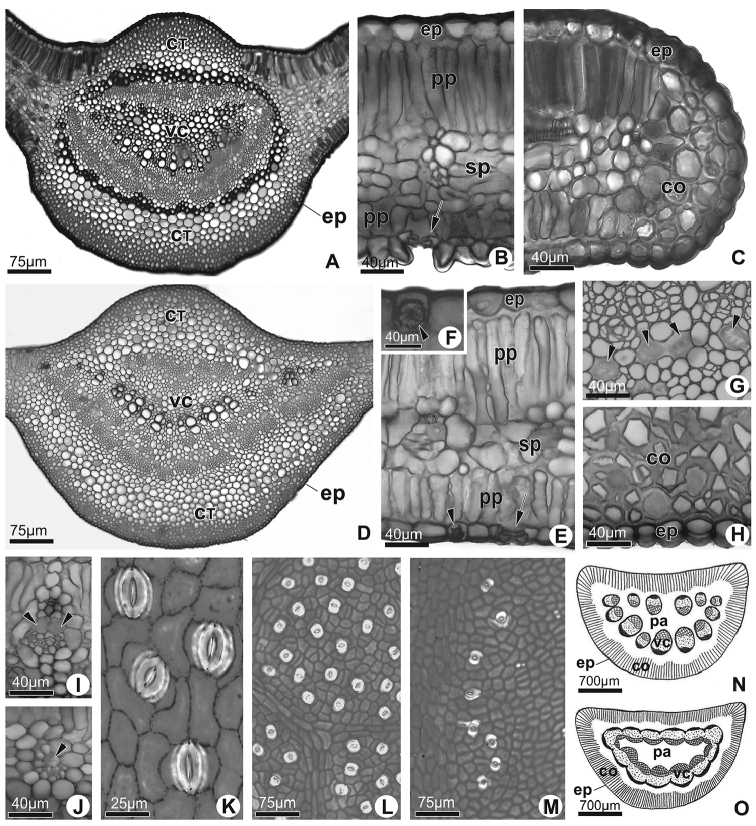
Leaf anatomy of species. **A–J** Cross section of the leaf blade. *Manihot fallax* (**A–C**): **A** Median portion of the midvein **B** Median portion of the leaf blade; abaxial epidermis papilose and stomata indicated by arrow **C** Edge. *Manihot attenuata* (**D–J**): **D** Median portion of the midvein **E** Median portion of the leaf blade; note stomata (arrow) and epidermal idioblast with druse (arrowhead) **F** Detail of epidermal idioblast with druse (arrowhead) **G** Laticifers in the midvein phloem (arrowheads) **H** Detail of the epidermis and collenchyma in the midvein **I** Vascular bundles with parenchymatical sheath in the median portion of the leaf blade (arrowheads correspond to laticiferous) **J** Vascular bundles in the median portion of the leaf blade (arrowheads correspond to laticifers) **K–M** Dissociated epidermis of both species. *Manihot fallax*. K. Paracytic stomata. *Manihot attenuata* (**L–M**): **L** Stomata distribution in the abaxial surface of the leaf blade **M** Stomata distribution in the adaxial surface of the leaf blade lateral to midvein **N** and **O** Schematic representation of the petiole in cross section **N** *Manihot fallax* **O** *Manihot attenuata*. CT: cortex; VC: vascular cylinder; co: collenchyma; ep: epidermis; pp: palisade parenchyma; sp: spongy parenchyma; pa: medullar parenchymatic.

Regarding leaf anatomical features, *M. fallax* differs from *M. attenuata* in having the vascular cylinder with arch-shaped collateral vascular bundles surrounded by pericyclic fibres in the midvein (Fig. 4A), uniseriate epidermis in the median portion of both surfaces of the leaf blade (Fig. 4B), at the edge (Fig. 4C) and petiole with collateral vascular bundles discontinuous in the vascular cylinder (Fig. 4N). In *M. attenuata*, the vascular cylinder has collateral vascular bundles in a flattened arc, not surrounded by pericyclic fibres in the midvein (Fig. 4D), epidermal cells without papillae in the median portion of the abaxial surface of the leaf blade (Fig. 4E), but with druses in the protoplast (Fig. 4E, F), as well as petiole with collateral vascular bundles continuous in the vascular cylinder (Fig. 4O).

Both *M. fallax* and *M. attenuata* have midveins with angular collenchyma on both surfaces, ground parenchyma surrounding the vascular cylinder (Fig. 4A, D, H), isobilateral mesophyll (Fig. 4B, E), edges with annular collenchyma (Fig. 4C), parenchymatic sheath surrounding the secondary veins towards the epidermal cells on both surfaces of the leaf blade (Fig. 4I) and other veins without sheath (Fig. 4J). Laticifers are also found in both species distributed along the phloem cells in the midvein, petiole and median portion of the leaf blade (Fig. 4G). Additionally, the petiole has epidermal cells and cortex similar to those of the midvein (Fig. 4N, O). The stomata are paracytic in both species (Fig. 4K), evenly distributed on the abaxial surface of the leaf blade (Fig. 4L) and in a parallel and continuous band on each side of the midrib (Fig. 4M). Due to the stomatal pattern, both species have amphistomatic leaves.

Amongst the anatomical features that differ between *M. fallax* and *M. attenuata*, the presence the fibres in the vascular cylinder in the midvein, the papillae in the epidermal cells and the arrangement and number of collateral bundles in the petiole have been cited by Allem (1984), Cunha Neto et al. (2014) and Graciano-Ribeiro et al. (2016) as characters that help differentiate species in *Manihot*.

### Manihot attenuata

Taxon classificationPlantaeMalpighialesEuphorbiaceae

Müll. Arg. (1874: 442) emend. M.J. Silva

Figs 5 , 6

Manihot brachystachys Pax & K. Hoffm., Planzenr. (Engler) 4, Fam. 147, II: 97. 1910. Type. Brazil. Südbrasilianishe Provinz, Staat Goiaz, 8 Jan 1898, fl., *Glaziou 22126* (lectotype, designated here: P [P04786133!]; isolectotypes: BR [BR000005101320!], G [G00441911!], K [000600417!], P [P04786134!, P04786135!], S [S-R-9076!]).
Manihot robusta M. Mend. & T. B. Cavalc. Arnaldoa 22(2): 297, 2015. Type. BRAZIL. Alto Paraíso de Goiás, Parque Nacional Chapada dos Veadeiros, ca. 0.3 km da GO-239, sentido sede alojamento do ICMBio (lado direito), 14°09'55.92"S, 47°47'25.62"W, 1046 m a.s.l., 31 Oct 2014, *M. Mendoza, J.B.A. Bringel, A.A. Santos and T. Reis 4343*, **syn. nov.** (holotype: CEN!; isotypes: HRCB!, HUEFS!, K!, MG, MO!, NY!, RB!, SP!, UB!).

#### Type.

BRAZIL. Goiás: inter Goyaz et Cavalcante, *Burchell 7865* (lectotype, designated here: K [K000600418!]; isolectotypes: BR [BR510866!], G!).

#### Emended description.

Shrubs 0.5–1.2 m tall erect or decumbent, in which case the stem to 0.6 m tall with lateral branches to 1.3 m long, monoecious, glabrous. Stems and branches angulose, glossy, brownish, the bark exfoliating when adult and purplish, greenish or combinations of these when young; latex yellow or clear. Stipules 10–12 × 0.5–0.1 mm, lanceolate, sparsely serreate, persistent; petiole 3–4 mm long, greenish. Leaves alternate, spiral, sparsely distributed along the branches, but more concentrated near apex; blades 17.5–24 × 4–4.5 cm, elliptic, widely elliptic, ovate-lanceolate, oblong-elliptic or sometimes lanceolate, entire, unlobed, non-peltate, base obtuse or attenuate, apex acute and mucronulate, chartaceous, glabrous on both surfaces, adaxial surface dark purple or greenish purple, abaxial surface glaucous to cinereous, the latter with a smooth wax pattern, venation camptodromous-brochidodromous, primary veins prominent on both surface, secondary veins impressed on both surfaces, diagonally arranged towards the midvein, all pinkish to purplish. Racemes 4.5–10.5 cm long, staminate or bisexual, solitary, terminal or arising from dichotomy of the branches, erect or pendent, congested, glabrous, angulate, glaucous to cinereous, waxy. Staminate flowers: buds 4–7 × 3–6 mm, widely ovoid, yellowish, without purplish pigmentation; bracts 10–15 × 3–7 mm, widely ovate, foliaceous, apex acuminate, margins sparsely and irregularly denticulate, persistent; bracteoles situated along the lower third up to half of the pedicel, 4–4.6 × 1–1.5 mm, ovate or ovate-laceolate, foliaceous, persistent, subopposite; pedicels 3–3.2 mm long, cylindrical, glabrous, light green; calyx 7–15 × 5–10 mm, campanulate, green-yellowish, without purplish pigmentation, shortly tomentose internally, lobes ovate-oblong with apex obtuse or rounded, base truncate; stamens 10, in two whorls of five, glabrous, the longer 7.5–7.6 mm long, the shorter 4.5–4.6 mm long, both thickened, anthers 3–4 mm long, oblong, bright yellow, dorsifixed; disc 10-lobed, intrastaminal, dark yellow. Pistillate flowers: buds 6–8 × 4–6 mm, ovoid, green-yellowish, without purplish pigmentation; bracts 5–9 × 1.5–5 mm long, widely ovate, entire, apex acuminate, margin irregularly serrate, glabrous, persistent; bracteoles 4–6 × 1–3 mm, oblong-elliptic, foliaceous, persistent, margin denticulate, apex acuminate, opposite along the lower third of the pedicel, glabrous; pedicels 4–5 mm long, cylindrical-clavate, glabrous, green; calyx 7–9 × 5–7 mm, campanulate, yellowish green, without purplish pigmentation, glabrous externally, shortly tomentose internally, lobes triangular, base truncate; ovary 5–7 × 2–3 mm, ovoid to oblong, glabrous, green, disc patelliform, lobed, yellow; styles 3, shortly united at the base, densely papillose lobed. Capsules 0.8–1.8 × 0.7–1.2 cm, oblong, light green, smooth, glabrous, without wings, waxy, dehiscence septicidal and loculicidal; columella (carpophore) persistent, 0.78–0.8 × 1.2–1.3 mm (width at narrowest point in middle), dilating to 2–2.2 mm at both tip and base, narrowly alate. Seeds 7–10 × 5–6 mm, oblong, dark grey or cinereous, with black spots; caruncle prominent, widely triangular with entire apex, cream.

**Figure 5.**
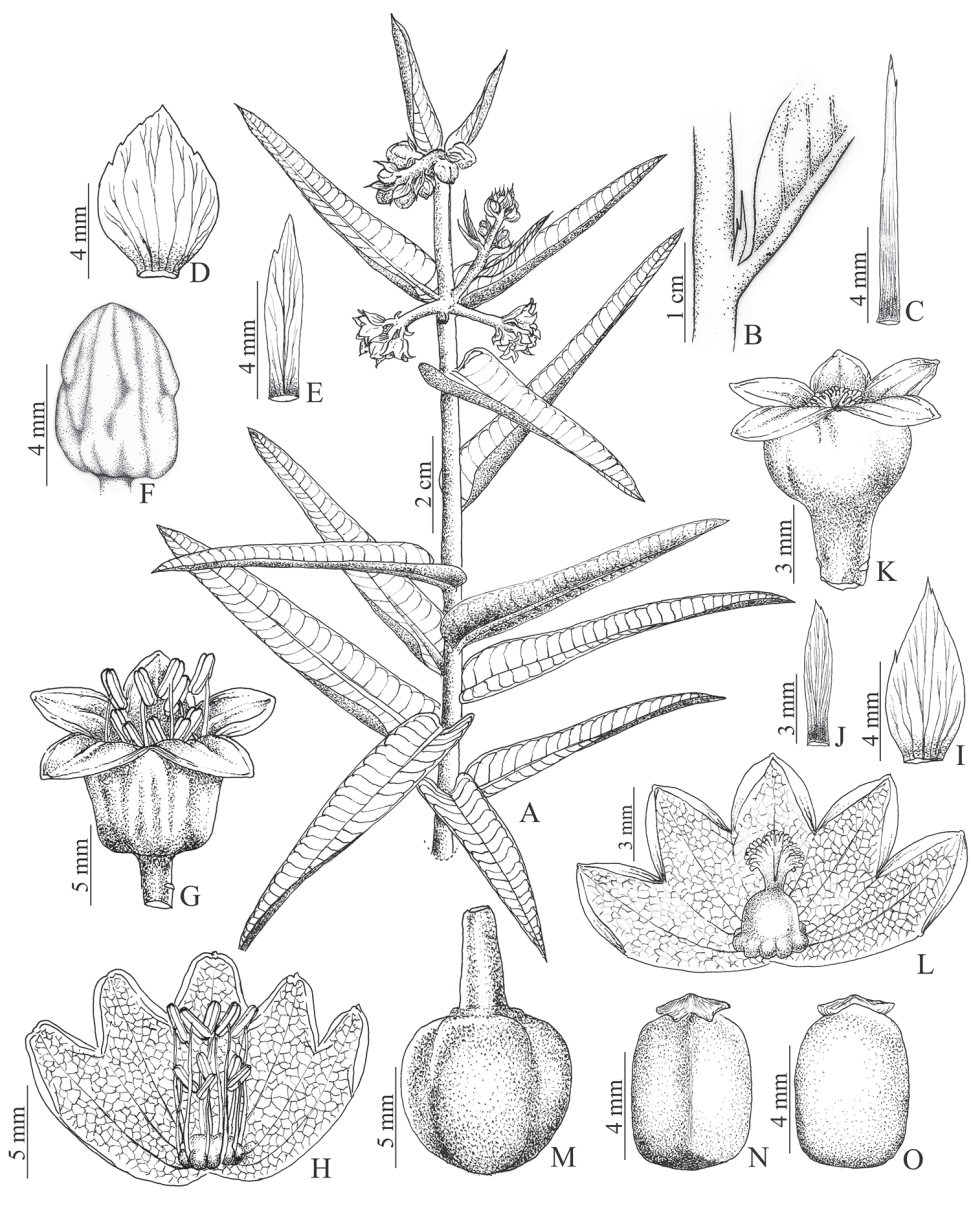
*Manihot attenuata*. **A** Habit **B** Details of the branch showing the persistent stipule **C** Stipule **D** Staminate bracts **E** Staminate bracteole **F** Staminate bud **G** Staminate flower **H** Staminate flower with calyx split and open **I** Pistillate bracts **J** Pistillate bracteole **K** Pistillate flower, note the gamosepalous calyx **L** Pistillate flower with calyx split and open **M** Fruit **N** Seed, ventral side **O** Seed, dorsal side. Drawn by Cristiano Gualberto from the holotype.

**Figure 6.**
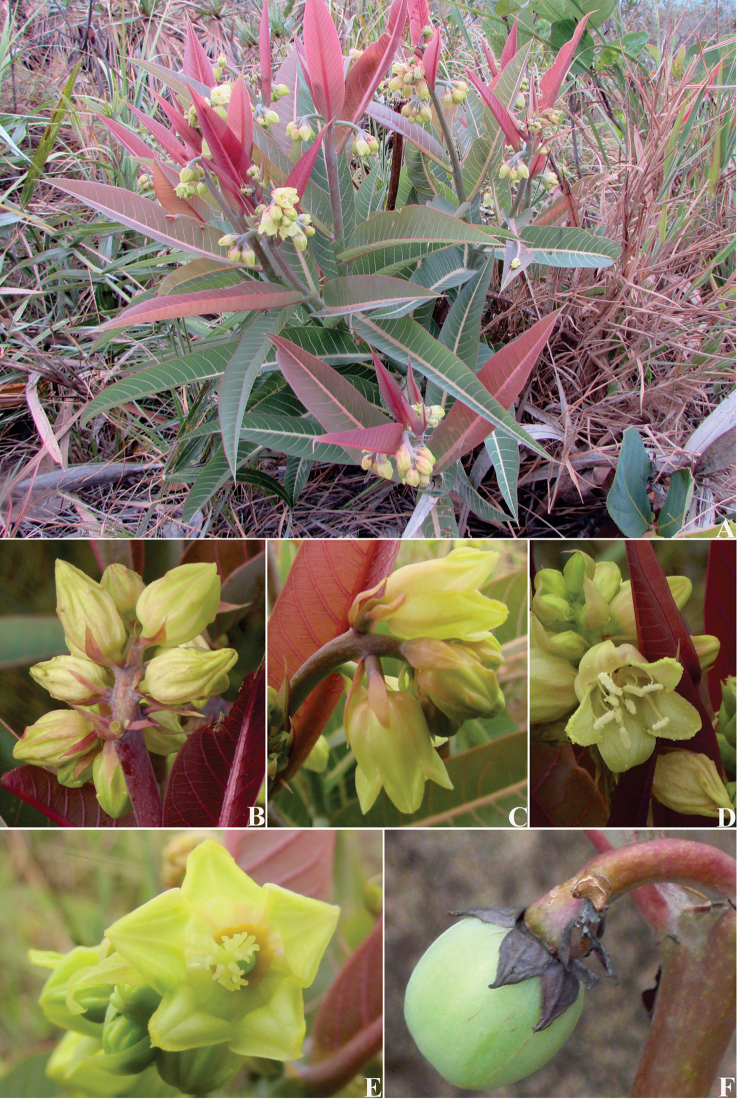
*Manihot attenuata*. **A** Habit **B** Inflorescence; note the ovoid staminate bud and showy bracts **C** Portion of inflorescence showing the staminate flowers, lateral view **D** Staminate flower, frontal view **E** Pistillate flowers in the inflorescence, frontal view; note the campanulate calyx **F** Fruit.

#### Morphological relationships and characterisation.

*Manihot attenuata* is easily recognised by its shrub habit; erect or decumbent habit; large leaves (17.5–24 × 4–4.5 cm) that are purplish; sparsely serrate, persistent stipules; bracts and bracteoles of both staminate and pistillate flowers widely ovate, pubescent externally and acuminate at the apex; usually pendent inflorescences; and pubescent stigmatic branches. It is morphologically similar to *M. fallax*. However, it differs by the set of characters cited in Table 1 and also according the anatomical characters previously discussed.

#### Distribution and ecology.

A species endemic to the northern portion of the state of Goiás, Chapada dos Veadeiros and neighbouring regions (municipalities of Niquelândia and Minaçu) (Fig. 3). It grows in cerrado rupestre, cerrado *sensu stricto*, near to rocky outcrops and in rocky fields, in flat sites, slopes or hilltops on clay-sandy soils, or in rock cracks, at altitudes from 440 and 1477 metres.

**Table 1.** Morphological characters useful in separating *M. fallax* from *M. attenuata*.

Character	*M. fallax*	*M. attenuata*
Leaves	8–14 × 1–2 cm, lanceolate, narrowly elliptic or linear, adaxial surface dark green, abaxial surface glaucous	17.5–24 × 4–4.5 cm, widely elliptic, ovate-lanceolate, oblong-elliptic or sometimes lanceolate, adaxial surface dark purple or greenish purple, abaxial surface glaucous or cinereous
Arrangement of secondary veins in relation to the midvein	Perpendicular	Diagonal
Stipules	Margin entire, early caducous 2.9–3 mm long	Margin serrulate, persistent, 10–11 mm long
Inflorescence	Racemes subspicate, 2.5–6 cm long	Racemes with well developed pedicels, 4.5–10.5 cm long
Bracts on flowers of both sexes	Glabrous, widely ovate with entire margin	Pubescent, widely elliptic with denticulate margin
Lobes of staminate calyx	Triangular with apex acuminate	Ovate-oblong with apex obtuse or rounded
Staminate buds	Obovoid	Ovoid
Filaments	Pubescent	Glabrous
Pistillate bracts	5–9 mm long, margin entire	6.4–7 mm long, margin irregularly serrate
Pistillate bracteoles	Lanceolate, margin entire	Oblong-elliptic, margin denticulate
Pistillate calyx	Dialisepalous	Gamosepalous
Caruncle shape	Triangular with bilobed apex	Widely triangular with entire apex

#### Phenology.

The species has been collected with flowers from November to March, with the flowers more common from September to December and with fruits from December to March.

#### Conservation Status.

*Manihot attenuata* is here classified as Vulnerable (VUVU) according to the IUCN Red List Categories and Criteria (IUCN 2014) because it has an extent of occurrence of ca. 9850 km^2^. However, the species has populations with more 20 individuals and it grows in environments inappropriate for farming and habitations, as well as in protected areas such as the Chapada dos Veadeiros National Park.

#### Typification.

**Figure 7.**
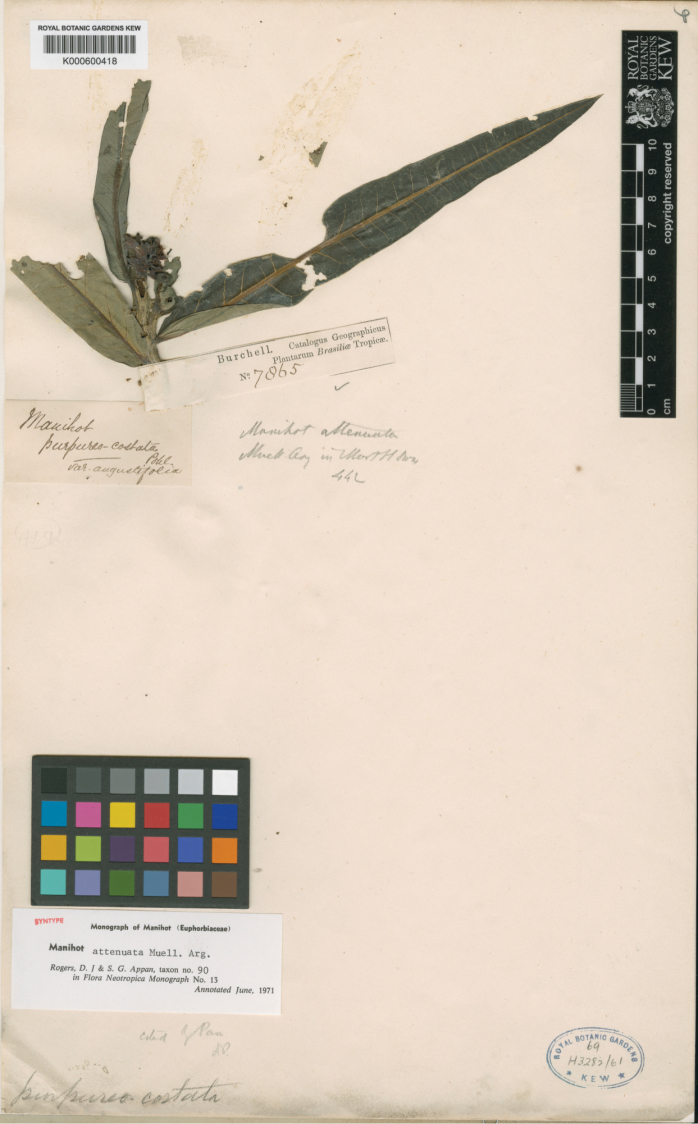
Specimen selected as the lectotype of *M. attenuata*, *Burchell 7865* (K000600418). Image used with permission and provided by the Royal Botanic Gardens, Kew.

Müller (1874) described *M. attenuata* in Flora Brasiliensis based on *Burchell 7865* from Goiás state. Pax (1910) recognised *M. attenuata* as a good species without comment about its typification and established *M. brachystachys*. Rogers and Appan (1973) subordinated *M. brachystachys* Pax & K. Hoffm. as a synonym of *M. attenuata* without comment or typification of the latter. Analysing all type collections of both species confirmed that both species need to be lectotypified. The collection *Burchell 7865* (Fig. 7) deposited in the herbarium K (K000600418) as lectotype of *M. attenuata* was designated here because it complies with the protologue and has flowers. Based on this same principle, the collection *Glaziou 21126* at P (P04786133) was proposed as a lectotype of *M. brachystachys*; isolectotypes are BR (BR0000005101320), G (G00441911), K (K000600417), P (P04786134, P04786135) and S (S-R-9076). *M. brachystachys* was also recognised as a synonym for *M. attenuata* because the characters used by Pax (1910) to differentiate them (e.g. leaf type, habit, fusion of floral parts) overlap and are variable within populations.

Recently, Mendoza et al. (2015) published *M. robusta* as a new species from Chapada dos Veadeiros region in Goiás state. Reviewing all collections used by the authors in the description of *M. robusta*, based on the images provided by them and the characters cited to differentiate *M. robusta* from *M. attenuata* (e.g. habit and aspect of growth, secondary vein numbers, inflorescence position and numbers, bract shape and length), it is concluded that the characters cited by the authors overlap and are variable within populations. Furthermore, all collections of *M. robusta* are from the same location as the type of *M. attenuata*. It is therefore considered that *M. robusta* is a synonym of *M. attenuata*.

#### Additional specimens examined.

**BRAZIL**. **Goiás**: Alto Paraíso de Goiás, Chapada dos Veadeiros, estrada GO 327, aproximadamente 1800 metros antes da Vila São Jorge, 14°09'00"S, 47°37'00"W, 1000 m a.s.l., 29 Mar 1988, *I. R. S. Costa et al. 19* (UFG); entrada do Parque Nacional da Chapada dos Veadeiros, 14°10'5"S, 47°47'25"W, 27 Jan 1996, fl., fr., *W. L. Werneck 706* (CEN); entrada do Parque Nacional da Chapada dos Veadeiros (a esquerda), estrada Alto Paraíso-Colinas, 34 km da GO 118, 14°09'49"S, 47°47'8"W, 1250 m a.s.l., 22 Jan 1997, fl., *B. M. T. Walter et al. 3644* (CEN); Parque Nacional Chapada dos Veadeiros (PNCV) área queimada com relevo plano, 15°44'14"S, 47°54'54"W, 1477 m a.s.l., 25 Sep 1995, fl., *M. L. Fonseca et al. 549* (IBGE); *ib.* Cerca de 1 km acima do alojamento dos brigadistas, 20 Jan 2012, fl. fr., *M. J. Silva et al. 4063, 4065* (UFG); *ib.*, Proximidades do alojamento dos brigadistas, 25 May 2012, fr., *L. C. S. Almeida et al. 42* (UFG); *ib.*, final do acesso da trilha que leva ao Cânion I, entre fendas de rocha, 14°09'6.55"S, 47°48'0.74"W, 1114 m a.s.l., 29 Sep 2012, fl., *M. J. Silva et al. 4421* (UFG); 2.5 km em direção ao rio Preto, 26 Oct 2012, fl., *M. J. Silva et al. 4502 and 4506* (UFG); na subida para o alojamento dos brigadistas do PNCV, 22 Oct 2011, fl., *M. J. Silva et al. 3885* (UFG); Parque Nacional Chapada dos Veadeiros, estrada que leva ao alojamento dos brigadistas, 14°9'53.6"S, 47°47'25.7"W, 1034 m a.s.l., 31 Oct 2014, fl., *L. S. Inocencio et al. 180, 181, 182* and *183* (UFG); *ib.*, segundo morro a nordeste do alojamento principal dos brigadistas do PNCV, 27 Oct 2012, fl., *M. J. Silva et al. 4503* and *4507* (UFG); topo da serra à direita da estrada que leva ao Cânion I, 14°09'05.7"S, 47°48'00"W, 1130 m a.s.l., 23 Nov 2012, fl., *L.C.S. Almeida & I. A. M. Watanabe 67* and *69* (UFG); estrada para Vale da Lua, 24 Nov 2012, fl., *M. J. Silva et al. 4589* (UFG); trilha a partir do dos alojamentos do PNCV em direção ao Cânion I, 14°08'S, 47°43'W, 1086 m a.s.l., 29 Nov 2012, fl., *L. C. S. Almeida et al. 50* (UFG); afloramento rochoso à nordeste do Cânion I, 14°9'S, 47°48'W, 1082 m a.s.l., 29 Nov 2012, fl., *L. C. S. Almeida et al. 51*, *52 and 56* (UFG); subida de acesso ao alojamento dos brigadistas do PNCV, 14°09'37.6"S, 47°57'2.3"W, 1034 m a.s.l., 22 Feb 2014, fl., fr., *M. J. Silva et al. 5921* (UFG); cerca de 500 m do alojamento dos brigadistas, 14°9'22.4"S, 47°47'38.9"W, 1089 m a.s.l., 13 Dec 2014, fl., *L. S. Inocencio et al. 278* (UFG); Parque Nacional da Chapada dos Veadeiros, estrada que leva ao alojamento principal do Parque, 04 Jan 2015, fl., *R. C. Sodré*, *M. J. Silva & C. H. G. M. Filho 1636* (UFG). Cavalcante, exatos 8 km a noroeste de Cavalcante ao longo da estrada de terra que leva à Colinas do Sul, 13°50'S, 47°33'W, 12 Dec 1986, fl., fr., *A. C. Allem et al. 11307* (CEN); estrada Minaçu-Cavalcante passando pela balsa Serra Branca (COTERRA), à cerca de 126 km do rio Tocantins, 13°41'07"S, 47°51'22"W, 740 m a.s.l., 10 Nov 2000, fl., *G. P. Silva 4421* (UFG); cerca de 4 km da Vila Veneno, na direção do rio São Félix, cerca de 12 km da Balsa da Coterra, 13°32'10"S, 48°03'29"W, 380 m a.s.l., 25 Jan 2001, fl., *B. M. T. Walter et al. 4807* (UFG). Chapada dos Veadeiros, imediações da Serra do Ministro, a aproximadamente 500 m da estrada entre Cavalcante e Colinas do Sul, 13°54'34"S, 47°38'55,4"W, 20 Jan 2014, fr., *R. C. Sodré et al. 1193* (UFG). Minaçu, 7 km após a entrada norte do canteiro de obras, 13°28'00"S, 48°23'00"W, 920 m a.s.l., 10 Mar 1992, fl., fr., *T. B. Cavalcanti et al. 1112* (CEN). Niquelândia, Reservatório em formação do AHE Serra da Mesa, região na margem direita do rio Maranhão, próximo ao rio das Almas, 14°34'50"S, 48°59'07"W, 440 m a.s.l., 16 Oct 1997, fl., *B. M. T. Walter 3924* (CEN).

## Supplementary Material

XML Treatment for Manihot fallax

XML Treatment for Manihot attenuata
